# Sequence Divergence and Conservation in Genomes of *Helicobacter cetorum* Strains from a Dolphin and a Whale

**DOI:** 10.1371/journal.pone.0083177

**Published:** 2013-12-17

**Authors:** Dangeruta Kersulyte, Mirko Rossi, Douglas E. Berg

**Affiliations:** 1 Department of Molecular Microbiology, Washington University Medical School, St Louis, Missouri, United States of America; 2 Faculty of Veterinary Medicine, University of Helsinki, Helsinki, Finland; University of Münster, Germany

## Abstract

**Background and Objectives:**

Strains of *Helicobacter cetorum* have been cultured from several marine mammals and have been found to be closely related in 16 S rDNA sequence to the human gastric pathogen H. pylori, but their genomes were not characterized further.

**Methods:**

The genomes of *H. cetorum* strains from a dolphin and a whale were sequenced completely using 454 technology and PCR and capillary sequencing.

**Results:**

These genomes are 1.8 and 1.95 mb in size, some 7–26% larger than *H. pylori* genomes, and differ markedly from one another in gene content, and sequences and arrangements of shared genes. However, each strain is more related overall to *H. pylori* and its descendant *H. acinonychis* than to other known species. These *H. cetorum* strains lack *cag* pathogenicity islands, but contain novel alleles of the virulence-associated vacuolating cytotoxin (*vacA*) gene. Of particular note are (i) an extra triplet of *vacA* genes with ≤50% protein-level identity to each other in the 5′ two-thirds of the gene needed for host factor interaction; (ii) divergent sets of outer membrane protein genes; (iii) several metabolic genes distinct from those of *H. pylori*; (iv) genes for an iron-cofactored urease related to those of *Helicobacter* species from terrestrial carnivores, in addition to genes for a nickel co-factored urease; and (v) members of the *slr* multigene family, some of which modulate host responses to infection and improve *Helicobacter* growth with mammalian cells.

**Conclusions:**

Our genome sequence data provide a glimpse into the novelty and great genetic diversity of marine helicobacters. These data should aid further analyses of microbial genome diversity and evolution and infection and disease mechanisms in vast and often fragile ocean ecosystems.

## Introduction

The genus *Helicobacter* consists of Gram-negative bacterial species that live in the gastrointestinal tracts of diverse animal hosts [1]–[3]. *H. pylori*, the best known of these species, chronically infects the gastric (stomach) mucosa of billions of people worldwide, is a major cause of peptic ulcer disease and gastric cancer, and is very diverse genetically. It is transmitted preferentially within families and local communities, apparently without major environmental reservoirs or alternate hosts [4]–[7].

Much less is understood about transmission and infection mechanisms, virulence, and population biology and evolution of other *Helicobacter* species. Although most of these species are known from land animals, a few also have been discovered in marine mammals. Of particular note is *H. cetorum* from marine mammals, defined to date primarily by its 16 S rDNA sequences [8]–[13], which are more closely related to those of *H. pylori* and the big cat pathogen *H. acinonychis* [14] than to those of other known species. PCR and 16 S rDNA sequence data indicate that *H. cetorum* is present in oceans worldwide [8]–[13], and suggest that it or close relatives also caused gastric infections in some urban Venezuelans [15] and lymph node infections in mule deer in Montana [16]. Interestingly, the genus *Helicobacter* belongs to the *Epsilonproteobacteria*, some of whose other members are associated variously with coral and sponge disease, and gastropods and biofilms of deep-sea hydrothermal vents [17]–[21]. Here, we sequenced the genomes of *H. cetorum* strains from a whale and a dolphin to help define this species' gene content and diversity, with long-range goals of better understanding pathogen transmission and infection mechanisms in marine ecosystems, genome evolution, and possible impacts of non-*pylori Helicobacter* species on animal and human health.

## Methods

### *H. cetorum* Culture and Genome Sequencing

The two *H. cetorum* strains that we sequenced had been cultured by Harper et al [8] from the main (glandular) stomach of a beached Atlantic white sided dolphin (MIT 99–5656, here called “dolphin strain”), and the feces of a captive (Mystic Aquarium) Beluga whale with esophageal and stomach ulcers (MIT 00-7128, here called “whale strain”), and had been deposited as ATCC BAA-540 and ATCC BAA-429 (or CCUG 52418 T), respectively [8]. The whale strain, although cultured from feces, was inferred to have lived in its host's stomach because its 16 S rDNA sequence was identical to that obtained by PCR from the animal's gastric tissue [8]. We grew these strains from single colonies using standard *H. pylori* culture conditions (BHI blood agar plates at 37°C, in 5% CO_2_, 10% O_2_ and 85% N_2_) and extracted genomic DNA as described [22], [23]. Genomic DNAs were sequenced using 454 FLX Titanium paired-end shotgun sequencing (>40-fold coverage), and reads were assembled using 454 Corporation Newbler software (164 and 88 contigs, dolphin and whale strains, respectively) by MOGene Corporation (St Louis, MO). We determined relative positions of contigs by PCR and filled all gaps between contigs by capillary sequencing of PCR products. The genome sequences were deposited in GenBank as accessions CP003481.1 (chromosome) and CP003482.1 (plasmid) of the dolphin strain, and NC_017737.1 (chromosome) and NC_017738.1 (plasmid) of the whale strain, and were annotated by the NCBI Prokaryotic Genome Automatic Annotation Pipeline staff, as described [23].

### Comparative Genomics and Phylogenetic Analysis

Complete, fully-annotated chromosome and plasmid sequences of the *Helicobacter* strains and species listed in Table 1 were downloaded from the NCBI ftp server; a database containing all predicted protein sequences was assembled and low-quality protein sequences were removed automatically. Reciprocal all-versus-all BLASTP was performed and results were processed by OrthoMCL using default parameters [24]. The OrthoMCL output was filtered using a perl script to produce different lists of ortholog groups (e.g. ortholog groups present in *H. cetorum* but not in *H. pylori*). Using the OrthoMCL output, we selected 126 genes in the core genome of gastric *Helicobacter* species with orthologs in a non-gastric outgroup species, *H. hepaticus* (Table S1). Alignments for each of these one-to-one rooted core genes were generated at the amino acid level using MAFFT-FFT-NS-i v.7 [25]; the proteins were back-translated to nucleotide sequence using Translatorx perl script [26]; aligned DNA sequences were concatenated using a perl script, and the phylogenetic tree was inferred using PhyML [27] by applying the following parameters: -b 2, -m GTR, -o tlr –a e, -c 6. A distance matrix of the concatenated aligned core genes was calculated using DISTMAT implemented in jEMBOSS using Kimura-2 [28].

**Table 1:** Strains and species used in this study.

	NCBI accession number	
Species and strain designation	Chromosomes	Plasmid
***Helicobacter cetorum***		
**MIT-00-7128**	NC_017737	NC_017738
***Helicobacter cetorum***		
**MIT-99-5656**	NC_017735	NC_017736
***Helicobacter pylori***		
**2017**	NC_017374	
**2018**	NC_017381	
**26695**	NC_000915	
**35A**	NC_017360	
**51**	NC_017382	
**83**	NC_017375	
**908**	NC_017357	
**Aklavik117**	NC_019560	NC_019561
**Aklavik86**	NC_019563	NC_019564
**B38**	NC_012973	
**B8**	NC_014256	NC_014257
**Cuz20**	NC_017358	
**ELS37**	NC_017063	NC_017064
**F16**	NC_017368	
**F30**	NC_017365	NC_017369
**F32**	NC_017366	NC_017370
**F57**	NC_017367	
**G27**	NC_011333	NC_011334
**Gambia94 24**	NC_017371	NC_017364
**HPAG1**	NC_008086	NC_008087
**HUP B14**	NC_017733	NC_017734
**India7**	NC_017372	
**J99**	NC_000921	
**Lithuania75**	NC_017362	NC_017363
**OK113**	NC_020508	
**OK310**	NC_020509	NC_020556
**P12**	NC_011498	NC_011499
**PeCan18**	NC_017742	
**PeCan4**	NC_014555	NC_014556
**Puno120**	NC_017378	NC_017377
**Puno135**	NC_017379	
**Rif1**	NC_018937	
**Rif2**	NC_018938	
**SJM180**	NC_014560	
**SNT49**	NC_017376	NC_017380
**Sat464**	NC_017359	NC_017356
**Shi112**	NC_017741	
**Shi169**	NC_017740	
**Shi417**	NC_017739	
**Shi470**	NC_010698	
**SouthAfrica7**	NC_017361	NC_017373
**UM032**	NC_021215	
**UM037**	NC_021217	
**UM066**	NC_021218	
**UM299**	NC_021216	
**XZ274**	NC_017926	NC_017919
**52**	NC_017354	
**v225d**	NC_017355	NC_017383
***Helicobacter acinonychis***		
**Sheeba**	NC_008229	NC_008230
***Helicobacter bizzozeronii***		
**CIII-1**	NC_015674	NC_015670
***Helicobacter heilmannii***		
**ASB1.4**	NC_019674	
***Helicobacter felis***		
**CS1**	NC_014810	
***Helicobacter mustelae***		
**12198**	NC_013949	
***Helicobacter hepaticus***		
**ATCC 51449**	NC_004917	

The two *H. cetorum* genome sequences were submitted to GGDC 2.0 [29], available at http://ggdc.dsmz.de, to calculate whole-genome distance and infer the degree of DNA-DNA hybridization between them.

To identify orthologs common to the two *H. cetorum* strains, the complete set of predicted proteins of one strain was compared with that of the other by reciprocal BLASTP. A BLAST score ratio cut-off of 0.4 was used to define two proteins as homologs.

Proteins identified by OrthoMCL as belonging to groups of orthologs that occur only in *H. cetorum* strains were then used as queries for BLASTP homology searches against the total NCBI database available in August 2013 to find related sequences, especially in *H. pylori*, and to better understand patterns of sequence conservation and divergence among related proteins.

## Results

### Phylogenetic Relationships of *H. cetorum* Strains

The chromosomes of the *H. cetorum* whale and dolphin and strains are 1.95 and 1.83 Mb Mb in size, respectively — a few hundred kb larger than is typical of *H. pylori* (1.55–1.71 Mb). Each strain also contains a plasmid, 12.5 and 14.1 kb in size, respectively (Table 2). The complete 16 S and 23 S rDNA sequences of these two strains differ by only 5 bp and 10 bp, respectively, and each is more closely related to the rDNAs of *H. pylori* and *H. acinonychis* than to those of other known species [8 and present results]. Whole genome BLASTN (http://blast.ncbi.nlm.nih.gov/) analyses confirmed and extended inferences from rDNA data — showing that these two strains are more closely related to various *H. pylori* strains or *H. acinonychis* than to any other known bacterial species. That said, only ∼64% of whale and ∼74% of dolphin strain genomes are found by BLASTN criteria in *H. pylori* genomes, and reciprocally, only ∼75–80% of representative *H. pylori* strain genome sequences are found in these *H. cetorum* genomes.

**Table 2:** General features of *H. cetorum* genomes.

Feature	MIT 00–7128, whale strain	MIT 99–5656, dolphin strain
**Chromosome**		
**Size bp**	1 947 646	1 833 666
**G+C content (%)**	34,5	35,8
**% Coding**	88	88,4
**Number genes**	1 775	1 731
**Protein coding**	1 731	1 689
**Structural rRNAs**	38	36
**16 S,23 S,5 S rRNAs**	2,2,2	2,2,2
***vacA***	one next to *cysS*	one next to *cysS,* plus three divergent between *ruvA, ruvB*
***cag*** ** pathogenicity island**	Absent	absent
**Urease**	two: nickel & iron co-factored	two: nickel & iron co-factored
**mobile DNAs**	two Tn*PZ* transposons; one near complete prophage with numerous rearrangements and insertions of probably non-phage DNAs	one IS*605*- and twenty IS*606*-like insertion sequences; one fragmented Tn*PZ* transposon; multiple and duplicated prophage fragments
**Plasmid**	one, pHCW	one, pHCD
**size (bp)**	12 465	14 124
**G+C content (%)**	34,5	32,7
**Number genes (orfs)**	13	15
**Other features**	putative replication and transfer genes also present in dolphin strain plasmid	putative replication and transfer genes also present in whale strain plasmid; two IS*606*, nearly identical to chromosomal IS*606*

**Figure 1:**
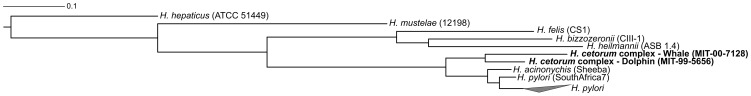
Phylogram representing maximum-likelihood tree of gastric *Helicobacter* species based on 126 aligned and concatenated core genes. The tree was inferred using PhyML applying General Time Reversible (GTR) model, estimating the gamma shape parameter by setting the number of substitution rate categories at 6. Statistical tests for branch support were conducted via a Chi2-based parametric approximate likelihood-ratio test (aLRT). All nodes are supported with aLRT values > 99%. The topology, branch lengths and rate parameters of the starting tree were optimized. The enteric (non-gastric) species *H. hepaticus* was used as outgroup. The core genes used for this figure are listed in Table S1.

The phylogenetic positions of these strains (Figure 1) were also inferred by Maximum Likelihood using 126 concatenated core genes (Table S1). All nodes in this tree are well supported with Chi2-based parameter branch values of over 99%. The two strains clustered together in the sister clade of *H. pylori/H. acinonychis*, but are separated by relatively long branches. The kimura-2 corrected distance value between these two strains, calculated based on these 126 core genes, is 16.15 substitutions per 100 bp (16%). Using these same core genes, the average distance between *H. pylori* or *H. acinonychis* and *H. cetorum* is approximately 20%, whereas that among sequenced *H. pylori* genomes is only 4.1%. Thus, at 16% substitution, these two *H. cetorum* strains differ from each other far more than would have been expected based on the near identity of their **16 S** rRNAs (1489/1494 bp).

**Figure 2:**
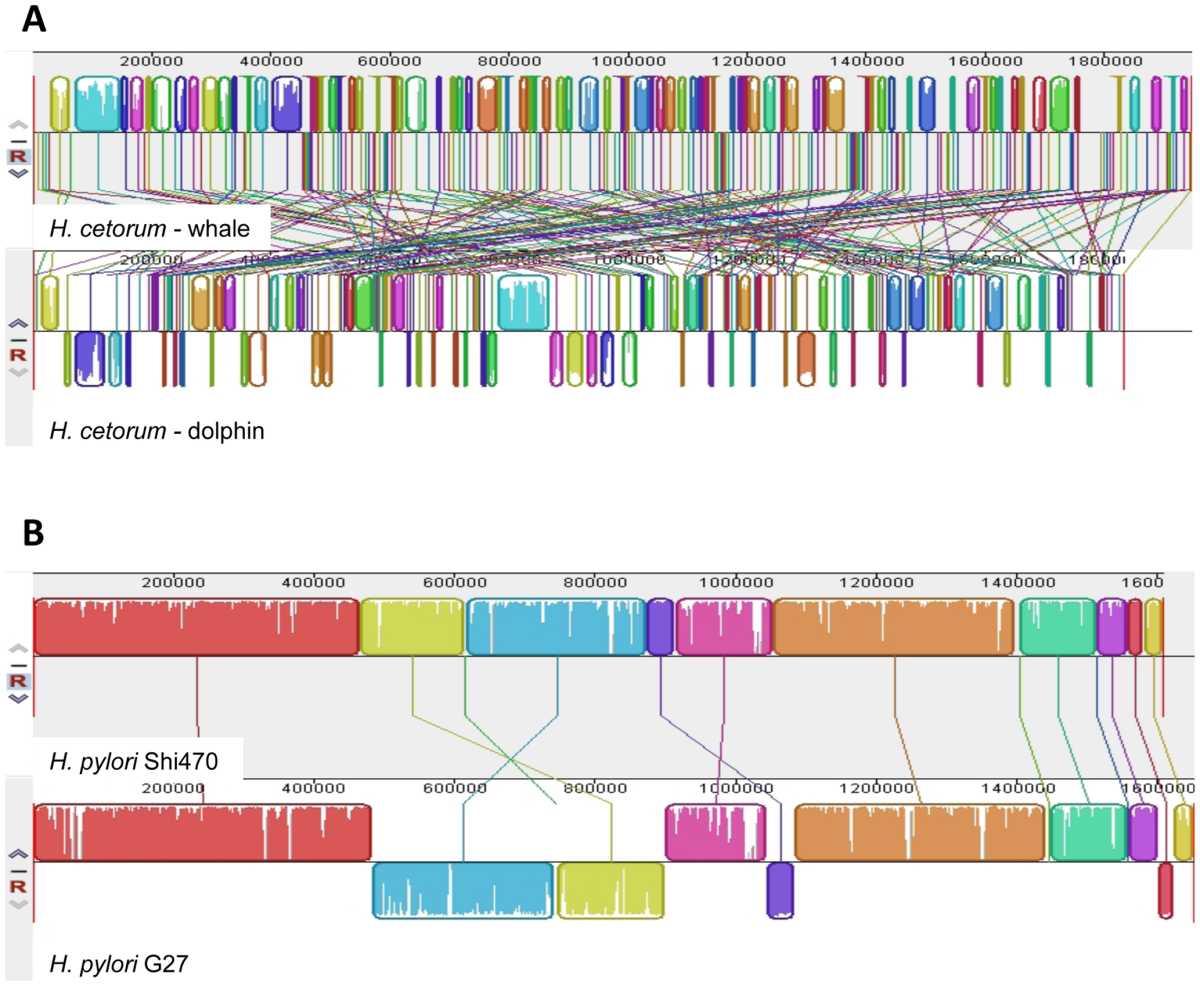
MAUVE alignment of representative *Helicobacter* chromosomes. For MAUVE software see http://gel.ahabs.wisc.edu/mauve/. A. Two *H. cetorum* genomes. B. Two representative *H. pylori* genomes. For further illustration of the higher conservation of gene order and orientation in *H. pylori* relative to *H. cetorum*, see [23].

Four additional tests were used to further characterize relationships of the *H. cetorum* strains to each other and to *H. pylori*, genome-wide. **First**, Mega BLAST analysis indicated that only 66% of dolphin strain DNA sequences are present in the larger whale strain genome. Similarly, BLASTN analysis of 1 kb chromosomal segments taken sequentially from the dolphin strain without regard to gene content indicated that some 30% of them have no significant homology to whale strain sequences. In contrast, pairs of *H. pylori* strains typically share >90% of chromosomal DNA sequences. The *H. cetorum* strain-specific DNAs are widely dispersed about their genomes, not concentrated in just one or a few sites (e.g., as chromosomal islands). **Second**, only 11% of sequential 1 kb chromosomal segments from the dolphin strain were at least 95% identical to whale strain sequences for at least 500 bp. In contrast, with even the least related pairs of *H. pylori* strains, ≥95% identities for >500 bp are found in more than 40% of such 1 kb segments. **Third**, chromosome alignment using MAUVE software revealed 204 differences in location and orientation of shared DNA segments between the *H. cetorum* strains (Figure 2A). In addition, the dolphin and whale strain chromosomes exhibited 135 and 203 differences, respectively, in DNA arrangement when aligned with that of a representative *H. pylori* strain (G27 [30]), whereas less than 10–15 DNA arrangement differences are found when comparing chromosomes of most other *H. pylori* strains with one another, as illustrated with strains G27 and Shi470 in Figure 2B [see also reference 23]. **Fourth**, DNA-DNA hybridization (DDH) parameters, estimated *in silico* by calculating whole-genome distance using the GGDC website, yielded a DDH estimate 29.1%±2.44 for these two strains. Based on conventional criteria [29], this indicates a probability via logistic regression of only 0.07% that they belong to the same species. A **fifth** test of relatedness and divergence emerged from our *in silico* proteome analyses, below.

### *In silico* Proteome Analysis

Examination of annotated genomes identified 86,309 predicted protein sequences in the chromosomes of 48 *H. pylori* strains and seven other *Helicobacter* species and in 25 *Helicobacter* plasmids (Table 1). Based on MCL clustering, 96% of the proteins were divided into 2,934 groups of orthologs (GOs), of which 1,478 and 1,434 GOs were detected in the whale and dolphin strain proteomes, respectively. Approximately 10% (164) of whale and 7% (112) of dolphin strain proteins have no orthologs in other genome sequenced *Helicobacter* species, and thus might be unique to *H. cetorum*. Among the 2,934 GOs, 157 are represented in whale but not dolphin strain proteomes, and 113 are represented in dolphin but not whale strain proteomes. The two *H. cetorum* strain proteomes were compared further using a BLAST score ratio cut-off of 0.4, which is more stringent than OrthoMCL, and can separate distant proteins that cluster in the same group by MCL. BLAST analysis identified 411 whale strain proteins (24% of proteome), with no significant homology to any dolphin strain protein, and conversely, 346 dolphin strain proteins (22% of proteome) with no significant homology to any whale strain protein. Thus, these data indicate considerable differences in the proteomes of these two *H. cetorum* strains.

### *H. cetorum*-specific Genes

**Figure 3:**
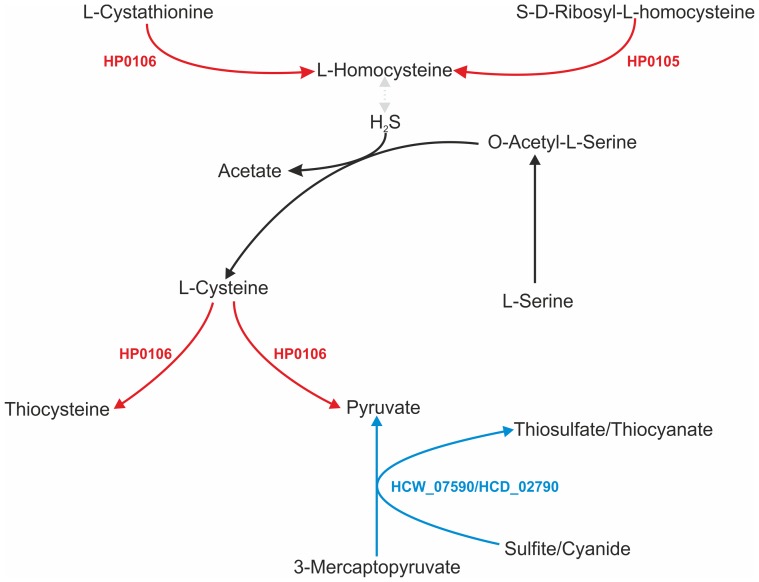
Schematic representation of cysteine and methionine metabolism (based on KEGG pathway 00270). In blue, reactions predicted in *H. cetorum* but not *H. pylori* with locus tags of the unique *H. cetorum* rhodanese-related sulfurtransferase gene indicated. In red, reactions predicted in *H. pylori* but not *H. cetorum*. In black, reactions predicted in both *H. cetorum and H. pylori*. A reaction for which no predicted enzymes were found in *Helicobacter* genomes is indicated by the dotted line and arrowheads in gray. Of note, DNA sequences matching those of HP1045 (acetyl CoA synthetase) are missing by BLASTN criteria from each *H. cetorum* strain, and also from 14 of the 48 fully sequenced *H. pylori* genomes screened. HP1045 was not included in Table 5 because of its absence from a significant minority of *H. pylori* strains.

**Figure 4:**
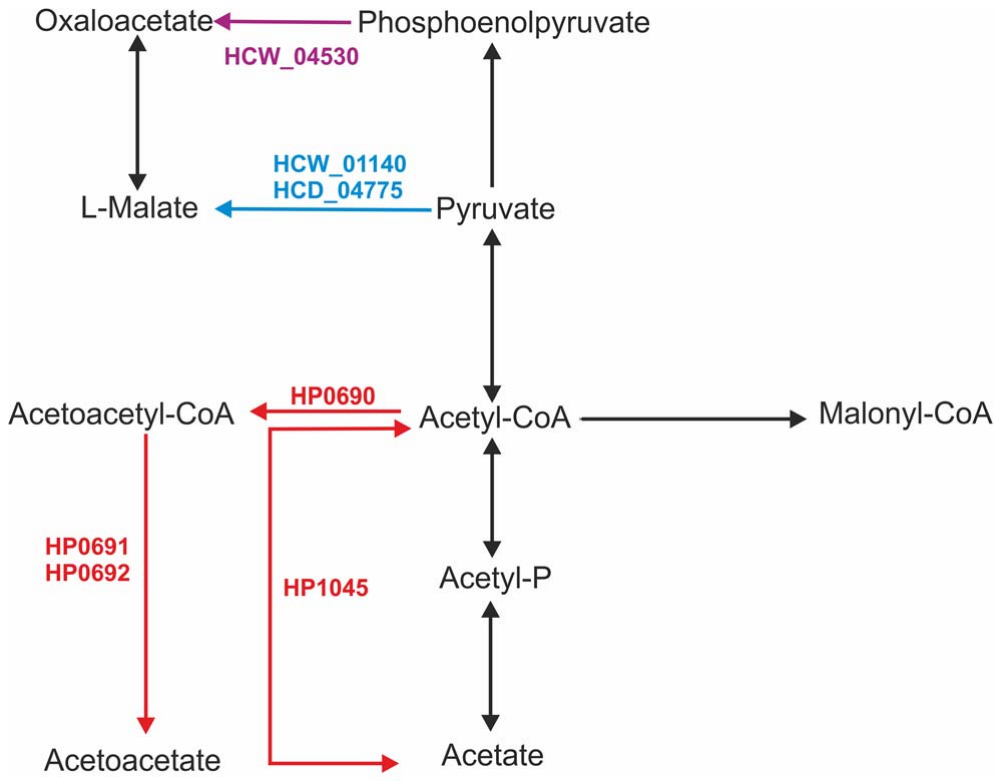
Schematic representation of pyruvate metabolism (based on KEGG pathway 00620). In blue, reactions predicted in *H. cetorum* but not *H. pylori*, with locus tags of the unique *H. cetorum* malate dehydrogenase indicated. In red, reactions predicted in *H. pylori* but not *H. cetorum*. In black, reactions predicted in both *H. cetorum and H. pylori*. In green, a reaction predicted only in the *H. cetorum* whale strain, not in the dolphin strain, nor in *H. pylori*.

Forty-six GOs were found in the two *H. cetorum* strains but not in any *H. pylori* strain (Tables 3 and 4) by initial OrthoMCL-based screening using the genome-sequenced strains listed in Table 1. Of particular interest are enzymes of central intermediary metabolism such as a rhodanese-related sulfurtransferase (HCW_07590, HCD_02790), which KEGG pathway analysis suggests could catalyze synthesis of pyruvate and thiosulfate from 3-mercaptopyruvate (Figure 3; blue arrows) or possibly other substrates. Homologous sulfurtransferases seem to be absent from nearly all other genome-sequenced *Epsilonproteobacteria*, including all other *Helicobacter* spp. and *Campylobacter* spp. A second example is that of the NADP-dependent malic enzyme (HCW_01140, HCD_04775), that could catalyze synthesis of L-malate from pyruvate (Figure 4, blue arrows). Related malic enzymes have been found in many extragastric *Helicobacter* spp. and in *Campylobacter* spp., but not in any *H. pylori* strain. Conversely, 22 GOs were detected in the *H. pylori/H. acinonychis* clade but not in *H. cetorum*, as illustrated in Table 5. We note, in particular, enzymes that could mediate synthesis of L-homocysteine, conversion of L-cysteine to thiocysteine or pyruvate (Figures 3, red arrows); and syntheses of acetoacetyl-CoA and acetate from acetyl-CoA, and of acetoacetate from acetoacetyl-CoA (Figure 4, red arrows). Finally, a phosphoenolpyruvate carboxylase that could catalyze oxaloacetate synthesis from phosphoenolpyruvate (Figure 4; light green arrow) is encoded in the genomes of the whale strain and of several other *Helicobacter* species, but not in the dolphin strain genome, nor in any *H. pylori* or *Campylobacter* strain genome sequenced to date.

**Table 3:** *H. cetorum* whale strain proteins distinct from those in *H. pylori* strains.

Locus Tag	GO	# amino acids (aa)	Annotation	Matches in *H. cetorum*, aa identity (blastp)	Matches in *H. pylori* aa identity (blastp)
**HCW_00105**	HEL3581	246	Hypothetical	HCD_03325, 54% in aa 68–139	None
**HCW_00115**	HEL3059	122	Hypothetical	HCD_03315, 89%	None
**HCW_00125**	HEL3852	246	HcpA	HCD_03275, 95%	Most strains, ≤32% in aa ∼97–241,
**HCW_00130**	HEL3853	421	Hypothetical	HCD_03280, 94%	None
**HCW_00595**	HEL3854	208	hypothetical, COG0500, SAM-dependent methyltransferase	HCD_03930, 98%	None
**HCW_01140**	HEL3270	420	Malate dehydrogenase	HCD_04775, 95%	None (1)
**HCW_01270**	HEL3858	290	COG0338, DNA adenine methylase	HCD_08595	Two strains, 65%, 72% (2)
**HCW_01595**	HEL3859	1437	COG3468 anticodon nuclease masking agent;	None	None (3)
**HCW_01740**	HEL3860	390	COG0477, drug transport transmembrane	HCD_00760, 98%	None
**HCW_02225**	HEL3057	752	OMP; pfam01856	HCD_02935, 54%; HCD_05585, 50%; HCW_07955, 32%; HCD_00325, 33%; HCD_08430, 31%; HCD_05575, 30% (4)	Many strains, ≤28%
**HCW_02500**	HEL3861	488 aa	Hypothetical	HCD_03555, 38% in aa 1–281, 61% in aa 285–488	None
**HCW_03170**	HEL3864	891	OMP, HomB, pfam01856	HCD_05580, 66%	Many strains, ≤31%
**HCW_03370**	HEL3867	73	copper binding, chaperone	HCD_06365, 60%	Many strains, ≤42% (5)
**HCW_03525**	HEL3071	211	Hypothetical	HCD_07120, 50%; HCD_07510, 51%; HCD_00640, 42%; HCD_01920,42%	Two strains, ≤46%
**HCW_04205**	HEL3869	341	Hypothetical	HCD_08400, 92%; HCD_02210, 75% in aa 182–301	None
**HCW_04215**	HEL3870	146	Hypothetical	HCD_08395, 97%	None
**HCW_04220**	HEL3871	155	Hypothetical	HCD_08385, 74%; HCW_05395, 89% in aa 1–64	Several strains, ≤79% in aa 1–62
**HCW_04245**	HEL3872	74	Hypothetical	HCD_08370, 93%	None
**HCW_04250**	HEL3873	113	Hypothetical	HCD_08365, 95%	None
**HCW_04255**	HEL3874	332	COG0582 integrase (6)	HCD_08360, 92%	Many strains, ≤39%
**HCW_04280**	HEL3061	291	Hypothetical	HCD_07575, 51% in aa 1–167	None
**HCW_04310**	HEL3062	72	Hypothetical	HCD_07600, 88%	None
**HCW_04320**	HEL3625	603	Hypothetical	HCD_07610, 79% in aa 1–100, 80% in aa 336–603	Many strains, ≤28% in N terminal, C sub-terminal domains
**HCW_04375**	HEL3875	69	Hypothetical	HCD_07645, 94%	None
**HCW_04395**	HEL3876	191	Hypothetical	HCD_07665, 83%	None
**HCW_04410**	HEL3877	111	Hypothetical	HCD_07680, 80%	Many strains, ≤27%
**HCW_04415**	HEL3878	237	Hypothetical	HCD_07685, 96%	Many strains, ≤29%
**HCW_04530**	HEL2846	870	phosphoenolpyruvate carboxylase	None	None (7)
**HCW_04560**	HEL3620	385	COG0286 type I restriction-modification HsdM	HCD_02110, 93%	Many strains, ≥38% in aa 65–383
**HCW_04565**	HEL3879	194	COG0732 type I restriction-modification HsdS	HCD_02105, 97%	None
**HCW_04635**	HEL3880	470	OMP_2, pfam02521	HCD_05480, 78%; HCW_06475, 40%	Many strains, ≤38%
**HCW_04920**	HEL2809	799	OMP, HopG, pfam01856	HCD_06965, 65% in aa 64–799; HCD_06965, 65%; HCW_06795, 99%; HCW_07665, 76%; HCW_06910, 52%; HCW_07970, in 482–799, 79% (4)	Many strains, ≤40% in aa 627–799
**HCW_05300**	HEL2800	446	OMP, pfam01856	HCD_06515, 51%; HCD_02735, 45%; HCD_00320, 44% (4)	None
**HCW_06445**	HEL3881	720	Type I restriction-modification HsdM	HCD_02745, 72%	None
**HCW_06450**	HEL3882	481	SSF116734, Type I restriction-modification HsdS	HCD_02740, 40%	None
**HCW_06795**	HEL2809	799	OMP, HopG, pfam01856	HCD_06965, 65% in aa 64–799; HCD_06965, 65%; HCW_04920, 99%; HCW_07665, 76%; HCW_06910, 52%; HCW_07970, in 482–799, 79% (4)	None
**HCW_06910**	HEL2809	718	OMP, HopG, pfam01856	HCW _04920 & _06795, 52%; HCD_06965, 52%; HCW_07970, 74% in aa 509–718 (4)	None
**HCW_07065**	HEL3884	419	OMP3	HCD_07105, 45%; HCD_08025, 61%; HCW_07110, 52% in aa 154–419; HCW_07075, 52% in aa 152–419	Many strains, ≤48% in aa 161–419
**HCW_07120**	HEL3885	128	hypothetical; CRISPR/Cas system associated	HCD_08225, 83%in aa 69–128	None
**HCW_07125**	HEL3886	274	CRISPR/Cas system-associated RAMP superfamily protein Cas6	HCD_08220, 91% in aa 1–192	None
**HCW_07130**	HEL3887	550	CRISPR/Cas system-associated protein Cas10	HCD_08210, 46% in aa 16–132; HCD_08205, 54% in aa 350–421	None
**HCW_07495**	HEL3889	1054	COG1002 type II restriction-modification. N-6 adenine methylase	HCD_01155, 90%	One strain, 77%
**HCW_07510**	HEL7510	210	Hypothetical	HCD_00640, 50%; HCD_07210, 43%; HCD_02790, 64%; HCW_03525, 51%; HCW_01920, 50%	Two strains, ≤53%
**HCW_07590**	HEL3071	413	COG2897 rhodanese-related sulfur transferase	HCD_02790, 64%;	None (8)
**HCW_07625**	HEL3891	180	Hypothetical	HCD_0820, 67%; HCD_08215, 25%	
**HCW_07630**	HEL3073	97	hypothetical; COG0790 FOG: Sel1-like repeat family	HCD_08525, 69%; HCW_07635, 75%	One strain; 50% in aa 59–88
**HCW_07635**	HEL3073	89	hypothetical; COG0790 FOG: Sel1-like repeat family	HCD_08525, 74%; HCW_07630, 75%	None
**HCW_07665**	HEL2809	810	OMP, HopG, pfam01856	HCD_06965, 66%; HCW_04920 & HCW_06795, 76%; HCW_06910, 52% in aa 97–810; HCW_07970, 43%(4)	Many strains; <38% in C terminal 120 aa
**HCW_07955**	HEL3892	731	OMP HomB	HCD_00325, 53%; HCD_02935, 33%; HCD_05585, 31%; HCD_03000, 30% in aa 13–503, 79% in aa 550–731; HCD_01075, 34% in aa 177–831; HCD_01075, 34% in aa 208–731; HCW_08600, 37%; HCW_02225, 32%	Many strains, ≤32% ident in aa 177–831
**HCW_08150**	HEL3894	117	Hypothetical	HCD_07625, 86%	None
**HCW_08195**	HEL3895	108	Hypothetical	HCD_08390, 83%; HCW_04200, 83%	None
**HCW_08600**	HEL3058	795	OMP; pfam01856	HCD_08430, 54%; HCD_03000, 54%; HCD_00325, 42%; HCD_05585, 32%; HCD_02935 in aa 184–795; HCD_01285, 30%; HCW_02225, 32% in aa 195–795	Many strains, ≤32% in aa 210–795

*Campylobacter* species.^(1)^ Homologs of HCW_01140 in many

% aa identity in many other *H. pylori* strains.^(2)^ Distant homologs of HCW_01270 with up to 29

*Helicobacter* species such as *H. bilis, H. winghamensis*, and *H. fennelliae*.^(3)^ Distant homologs of HCW_01595 in other

% and 50% identity overall to HCD_02935 and HCD_05585, but >85% identity to these proteins starting at aa position ∼590 of the 752 aa long protein.^(4)^ For most OMPs in this table, distribution of identities throughout protein is distinctly non-random, with highest sequence conservation in carboxy terminal, and in some cases also amino terminal domains. For example, HCW_02225 exhibits 54

% aa identity in other *Helicobacter* species including *H. cinaedi*, *H. bizzozeronii*, and *H. hepaticus*.^(5)^ Homologs of HCW_03370 with up to 39

^(6)^ HCW_04255 is just one of four "integrases" annotated in the whale strain proteome.

–54% in H. bizzozeronii, H. felis, H. bilis, H. fennelliae, H. mustelae, H. hepaticus and Wolinella.^(7)^ Homologs of HCW_04530 with identities of 47

–38% in multiple strains of *Leptotrichia, Actinobacillus, Providencia, Haemophilus, Morganella*, etc.^(8)^ Homologs of HCW_07590 with aa identities of 35

**Table 4:** *H. cetorum* dolphin strain proteins distinct from those in *H. pylori* strains.

Locus Tag	GO	# amino acids (aa)	protein annotation	Matches in *H. cetorum*, aa identity (blastp)	Matches in *H. pylori*, aa identity (blastp)
**HCD_00320**	HEL2800	487	OMP, HopK, pfam01856	HCW_05300, 50% in aa 182–487; HCD_08540, 37% in aa 23–367; HCD_02735, 55%; HCD_06515, 51%;	Many strains, ≤37% in aa 330–465
**HCD_00325**	HEL3892	741	hypothetical	HCD_03000 & _08430, 47%; HCD_05585, 31%; HCW_07955, 53%; HCW_08600, 42%	Many strains, ≤31%
**HCD_00760**	HEL3860	396	COG0477, sugar/drug transport membrane	HCW_01740, 98%;	None
**HCD_01155**	HEL3889	1054	Type II restriction-modification, N-6 adenine methylase	HCW_07495, 90%	One strain, 78%
**HCD_02105**	HEL3879	194	COG0732 Type I restriction-modification. HsdS	HCW_04565, 97%;	Many strains, ≤25%
**HCD_02110**	HEL3620	385	COG0286 Type I restriction-modification. HsdM	HCW_04560, 93%	Many strains, ≤37%
**HCD_02735**	HEL2800	506	OMP, HopK	HCD_08540, 100% in aa 58–336; HCD_06515, 58%; HCD_00320, 55%	Many strains, ≤37% in aa 306–506
**HCD_02740**	HEL3882	498	SSF116734: Type I restriction modification. DNA specificity domain superfamily HsdS	HCD_06450, 40%	Many strains, ≤30% ident for <∼200 aa from many parts of protein
**HCD_02745**	HEL3881	720	COG0286 Type I restriction-modification. N-6 adening methylase HsdM	HCD_06445, 72%	Many strains, ≤24% identity, C terminal ∼half of protein
**HCD_02790**	HEL3890	403	COG2897 rhodanese-related sulfur transferase	HCW_07590, 64%	None (1)
**HCD_02935**	HEL3057	746	OMP, pfam01856	HCW_02225, 55%; HCW_07955, 34%; HCW_08600, 34% in aa 135–746; HCW_03765, 29% in aa 197–746; HCW_02225, 55%; HCD_05585, 55%; HCD_00325, 34%; HCD_05575, 32%	Many strains, ≤28%
**HCD_03000**	HEL3058	806	OMP, HomB, pfam01856	HCW_08600, 54%;HCW_07955, 39%; HCD_08430, 78%; HCD_00325, 46%; HCD_05585, 31%; HCD_01285, 30%	Many strains, ≤31% in aa 213–806
**HCD_03265**	HEL3059	122	hypothetical	HCW_00115, 89%; HCD_03315, 100%.	None (2)
**HCD_03275**	HEL3852	248	HcpA, cysteine rich protein	HCW_00125, 95%	All strains, 32% in aa 93–241
**HCD_03280**	HEL3853	274	hypothetical	HCW_00130, 94%	None
**HCD_03315**	HEL3059	122	hypothetical	HCW_00115, 89%; HCD_03265, 100%	None (2)
**HCD_03325**	HEL3851	72	hypothetical	HCW_00105, 54%	None
**HCD_03555**	HEL3861	575	hypothetical	HCW_02500, 40% in aa 1–308 & 61% in aa 410–575 (deletion, codons 309–409)	None
**HCD_03930**	HEL3854	208	hypothetical, COG0500 SAM-dependent methyltransferase	HCW_00595, 98%	None
**HCD_04775**	HEL3270	422	NADP-dependent malic enzyme	HCW_01140, 95%	None (3)
**HCD_04915**	HEL3859	63	anti-codon nuclease masking agent (fragment of >1400 aa protein)	HCW_01595, 68% (match to internal segment)	None
**HCD_05580**	HEL3864	675	OMP, HomB, pfam01856	HCW_03170, 66%; HCW_01770, 31%; HCW_06190, 32%; HCW_03165, 32%; HCD_01070, 31%; HCD_00840, 32%; HCD_05575	Many strains, ≤32%
**HCD_05585**	HEL3057	784	OMP, pfam01856	HCW_02225, 50%; HCW_07955, 33%; HCW_08600, 32%; HCD_02935, 55%; HCD_00325, 33%; HCD_08430, 32%; HCD_03000, 32%; HCD_01075, 31%	Many strains, ≤28%
**HCD_05840**	HEL3880	483	OMP-2, pfam02521	HCW_04635, 80%; HCW_06475, 39%; HCW_04640, 36%; HCW_06805, 39%; HCW_05840, 38%; HCW_05835, 34%; HCW_03775, 32%; HCW_04625, 31%; HCD_05570, 39%; HCD_06420, 39%; HCD_05545, 37%; HCD_05845, 34%; HCD_07420, 32%; HCD_05850, 32%; HCD_08485, 31%; HCD_05830, 30%	Many strains, ≤39%
**HCD_06365**	HEL3867	73	COG2608, copper (metal) binding, chaperone	HCW_03370, 60%; HCW_03375, 42%; HCD_06360, 43%	Many strains, ≤45%
**HCD_06515**	HEL2800	473	OMP, HopK, pfam01856	HCW_05300, 52% in aa 151–473; HCD_08540, 45% in aa 47–353; HCD_02735, 58%; HCD_00320, 51%	Many strains, ≤35% in aa 281–473
**HCD_06965**	HEL2809	757	OMP, HopF, pfam01856	HCD_07970 54% in aa 279–834; HCD_02585, 52% in aa 584–757; HCW_07665, 66%; HCW_04920, 65%; HCW_06795, 65%; HCW_06910, 52%	Many strains, ≤42% in aa 564–757
**HCD_07210**	HEL3071	207	hypothetical	HCW_03525, 50%; HCW_01920, 40%; HCW_07510, 43%; HCD_00640, 41%	Two strains, 42%; and 45% in aa 88–207
**HCD_07600**	HEL3062	72	hypothetical	HCW_04310, 88%	None
**HCD_07610**	HEL3625	434	hypothetical	HCW_04320, 80%, but 603 aa (has internal replacement of 67 by 235 aa)	Many strains, ≤35%, most from aa 36 or 97 to aa 315
**HCD_07625**	HEL3894	108	hypothetical	HCW_08150, 80%	None
**HCD_07645**	HEL3875	69	hypothetical, type III restriction	HCW_04375, 94%	Many, ≤43% in aa 19–58
**HCD_07680**	HEL3876	110	hypothetical, COG0841,cation efflux, TrBC2/VirB2 family	HCW_04410, 80%	
**HCD_07685**	HEL3878	237	hypothetical	HCW_04415, 96%	None
**HCD_08025**	HEL3884	375	OMP-3	HCW_07065, 61%; HCW_07110, 45% in aa 125–375; HCW_07075, 50% in aa 127–375; HCW_07105, 33%; HCW_07115, 36% in aa 83–328; HCW_04520, 31%; HCD_02500, 41% in aa 127–375;	Many strains, ≤45% in aa 127–375
**HCD_08210**	HEL3887	133	CRISPR/Cas system protein Cas10	HCW_07130, 46%	None
**HCD_08220**	HEL3886	195	CRISPR/Cas system RAMP superfamily protein Cas6	HCW_07125, 91%	None (4)
**HCD_08225**	HEL3885	60	CRISPR/Cas system protein	HCW_07120, 83% (aa 69–128 of 128 aa long protein)	None (5)
**HCD_08310**	HEL3062	72	hypothetical	HCW_04310, 88%	None
**HCD_08345**	HEL3061	242	hypothetical	HCD_07575, 100%; HCW_04280, 51% in aa 1–154 (167 aa long protein)	None
**HCD_08360**	HEL3874	332	integrase	HCW_04255, 92%	Many strains, ≤38%
**HCD_08365**	HEL3873	113	hypothetical	HCW_04250, 95%	None
**HCD_08370**	HEL3872	74	hypothetical	HCW_04245, 93%	None
**HCD_08385**	HEL3871	153	hypothetical	HCW_04220, 74%; HCW_05395, 78% in aa 11–65	Two strains, 80% in aa 5–63
**HCD_08390**	HEL3872	108	hypothetical	HCW_08195, 83%; HCW_04200, 82%	None
**HCD_08395**	HEL3870	147	hypothetical	HCW_04215, 97%	None
**HCD_08400**	HEL3869	340	hypothetical	HCW_04205, 92%; HCW_02210, 75% in aa 182–299 (127 aa protein)	None
**HCD_08430**	HEL3058	812	OMP, HomB, pfam01856	HCW_08600, 54%; HCW_07955, 39%; HCD_03000, 79%; HCD_00325, 47%; HCD_01285, 31%	Many, ≤33% in aa 216–812
**HCD_08520**	HEL3891	179	hypothetical	HCW_07625, 67%; HCD_03555, 31% in aa 70–178	None
**HCD_08525**	HEL3073	58	COG0790 FOG Sel1 repeat c102723	HCW_07635, 74% and HCW_07630, 69% in aa 4–58. Homologs have 17 and 34 aa N-terminal extensions	None
**HCD_08540**	HEL2800	331	membrane, protein export, secD	No close HCW homolog. HCD_02735, 100% in aa 2–300; HCD_06515, 47% in aa 2–330; HCD_00320, 37% in aa 1–330	None
**HCD_08595**	HEL3858	291	COG0338 DNA adenine methylase	HCW_01270, 89%;	Several strains with aa identities of 31%–71%

–40% in multiple strains of *Actinobacillus, Leptotrichia, Haemophilus, Morganella, Providencia*, etc.^(1)^ Homologs of HCD_02790 with aa identities of 35

*H. felis, H. bizzozeronii*, and *H. fennelliae*.^(2)^ Distant homologs of HCD_03265 and HCD_03315 in

*Campylobacter* strains.^(3)^ Homologs of HCD_04775 in many

*H. pullorum*, *H. cinaedi* and *Campylobacter gracilis*.^(4)^ Homologs of HCD_08220 in several species including

*Campylobacter* and *Helicobacter* species.^(5)^ Homologs of HCD_08225 in several

**Table 5:** *H. pylori* strain 26695 proteins(1) belonging to 22 GOs in *H. pylori/H. acinonychis* clade not in *H. cetorum*.

*H. pylori* 26695 Locus_tag(1)	GO	NCBI annotation (*H. pylori* 26695)
**HP0085**	HEL2215	Hypothetical protein
**HP0092**	HEL1980	Type II restriction enzyme M protein (HsdM)
**HP0104**	HEL2216	2′,3′-cyclic-nucleotide 2′-phosphodiesterase
**HP0105**	HEL2077	S-ribosylhomocysteinase (LuxS)
**HP0106**	HEL2078	Cystathionine gamma-synthase/cystathionine beta-lyase (MetB
**HP0309**	HEL2219	N-carbomoyl-D-amino acid amidohydrolase (2)
**HP0311**	HEL2220	Hypothetical protein
**HP0312**	HEL2221	ATP-binding protein
**HP0338**	HEL2222	Hypothetical protein
**HP0614**	HEL2224	Hypothetical protein
**HP0630**	HEL2096	NAD(P)H-quinone reductase (MdaB)
**HP0690**	HEL2098	Acetyl Co A acetyltransferase
**HP0691**	HEL2099	Succinyl-CoA-transferase subunit A
**HP0692**	HEL2191	Succinyl-CoA-transferase subunit B (3)
**HP0696**	HEL2100	Acetone carboxylase alpha subunit
**HP0697**	HEL2226	Acetone carboxylase gamma subunit
**HP0730**	HEL2227	membrane protein (2)
**HP0851**	HEL2107	Pap2-like membrane protein (2)
**HP0871**	HEL2229	CDP-diacylglycerol pyrophosphatase
**HP0879**	HEL2230	Putative nuclease (2)
**HP0935**	HEL2200	Putative N-acyltransferase (2)
**HP1177**	HEL1225	Outer membrane protein (HopQ)
**HP1185**	HEL2045	Sugar efflux transporter

^(1)^ Gene names from original (1997) genome sequence deposition (NC_000915.1). The NCBI database also contains a recent deposition of a separately determined 26695 genome sequence with entirely different gene numbers (CP003904.1).

^(2)^ Designated as hypothetical in original 1997 publication; the function indicated here was suggested by other groups analyzing corresponding sequences in other strains.

*H. pylori* genomes inspected (Table 1), although its protein product was not identifiied in annotations of Shi417 and XZ274 because of apparent frameshift or nonsense mutations, which we suspect may result from DNA sequencing errors.^(3)^ The HP0692 gene sequence is present in all

Also of note are *H. cetorum* genes for an integrase, DNA restriction-modification, CRISPR/cas (anti-phage defense) systems, and metal (copper) binding, and numerous outer membrane proteins (OMPs; discussed further below) (Tables 3 and 4). For some of these, no homologs at all are found by BLASTP analyses in current *H. pylori* sequence databases. Many of the OMPs, however, are mosaic, with some segments well matched to those in *H. pylori* next to segments that are so divergent that we postulate functional differences, e.g., in their molecular or host cell targets or interaction partners. We suggest that many of the present strain-specific *H. cetorum* genes or gene fragments had been transferred from unrelated phyla, and that *Helicobacter* spp. adaptation to particular hosts can involve acquisition or loss of specific metabolic pathways, as was suggested during *H. bizzozeronii* genome analysis [31].

### Genes Likely to be Involved In Bacterial-Host Interaction

Genes implicated in bacterial host interactions and that differ markedly between *H. cetorum* and *H. pylori*, that are absent from *H. cetorum*, or that are present in *H. cetorum* but not *H. pylori* merit special attention.

#### vacA

**Figure 5:**
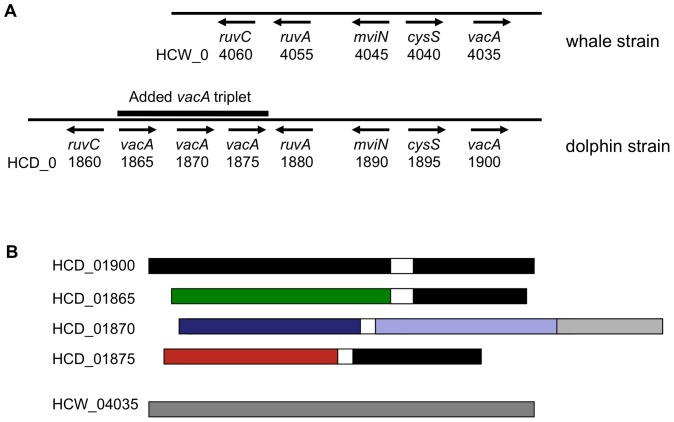
vacuolating cytotoxin (*vacA*) genes of *H. cetorum*. A, Chromosomal region containing *vacA* genes from the *H. cetorum* whale and dolphin strains. Arrows indicate gene orientation. B, Sequence conservation and divergence among *vacA* genes of *H. cetorum*. Lighter and darker shades of same color indicate ≥60% identity by BLASTP criteria. Completely different colors (black, green, blue, red) indicate ≤51% identity. To illustrate, amino acids (aa) 130–881 of gene HCD_01900 (*vacA* at normal location next to *cysS*) exhibit 40%, 50% and 65% identity to corresponding regions of HCD_01865, HCD_01875 and HCW_05035, respectively, and also 34–46% identity to corresponding regions of HCD_01870 (which itself has an internal divergent duplication with aa 1–694, just 67% identical to aa 734–1428). In contrast, aa 920–1342 of HCD_01900 exhibit 99% identity to corresponding carboxy terminal regions of HCD_01865 and HCD_01875, although only 58% and 69% identity to corresponding regions of HCD_01870 and HCW_04035. Similarly, the amino terminal ∼720 aa of HCD_01865 and HCD_01875 are each ≤50% identical to corresponding regions of other VacA proteins, whether from *H. cetorum* or *H. pylori*.

*H. pylori* strains encode a potent vacuolating cytotoxin (VacA) that contributes to bacterial fitness and can cause multiple structural and functional changes in host tissues — prominent among them, formation of anion-selective channels and cytoplasmic vacuoles, increased permeability of cell monolayers and mitochondrial membranes, and interference with antigen presentation, inflammatory responses and immune cell activation and proliferation [32]–[35]. To our knowledge, no intact *vacA* genes have been found in species other than *H. pylori. vacA* sequences are found in *H. acinonychis*, but only as fragmented pseudogenes in each of the several strains examined [14], [36]). In contrast, the two *H. cetorum* strains each contain intact *vacA* homologs next to *cysS*, the location also occupied in *H. pylori* (HCD_01900, 1342 codons, and HCW_04035, 1316 codons, in dolphin and whale strains, respectively). These *H. cetorum vacA* genes exhibit only 60%–68% protein-level identity to their most closely related *H. pylori* homologs, and only ∼66% identity to one another (Figure 5).

The dolphin strain contains, in addition, an extraordinary extra triplet of contiguous but divergent *vacA* genes (HCD_01865, HCD_01870, HCD_01875) inserted 6.5 kb from the *cysS*-linked *vacA* gene (HCD_01900) between two DNA repair/recombination genes, *ruvA* and *ruvC*, which are adjacent to one another in the whale strain (Figure 5A) (and curiously, adjacent or very near to one another in six of 16 genome sequenced *H. pylori* strains screened, including four strains from Africa). The dolphin strain's four *vacA* genes exhibit only 40% to 51% protein level identity to one another in the first ∼700–800 codons, a region important for VacA protein's secretion and multiple host cell intoxication functions [32]–[35]. In contrast, the protein from the first and third triplet members and the *cysS*-linked gene are 99% identical to one another in the last ∼340 amino acids (which determine VacA's autotransporter activity), but these well matched sequences are only 70% identical to the corresponding segment from the second member of the triplet (HCD_01870). The second triplet member's protein also contains an unusual divergent duplication of nearly 700 amino acids whose two components are only 67% identical to one another (Figure 5). The *vacA* triplet members each seem to lack ≥80 codons corresponding to 5′-ends of typical toxigenic *H. pylori* homologs (Figure 5) and thus may not be functional. Nevertheless these extra genes may contribute novel sequences and functionalities to other *vacA* genes by intragenic recombination. Just how these various *vacA* alleles affect the transport, actions and interactions of their encoded proteins, and bacterial virulence, host range and host responses to infection all merit further study.

*H. pylori* strains typically contain several genes annotated as toxin-like or *vacA*-like because the C-terminal autotransporter domains of their encoded proteins exhibit ∼30% identity to that of VacA. The *H. cetorum* strains also contain several such toxin-like genes, including one with ≥65% protein-level identity to *H. pylori imaA* (HP0289), found recently to help modulate host inflammatory responses to infection [37].

#### *cag* PAI and adjacent HP0159 gene

Each *H. cetorum* strain lacks a *cag* pathogenicity island (*cag* PAI), a ∼30 kb DNA segment present in more than half of *H. pylori* strains worldwide that is a major contributor to infection-associated inflammation and changes in epithelial structure and development, and that is disease-associated epidemiologically and a contributor to *H. pylori* fitness and virulence in cell culture and animal infection models [38]–[42]. Also absent is a close homolog of gene HP0519, which is next to one *cag* PAI end in *cag*-positive *H. pylori*, seems to have undergone intense selection for amino acid sequence change in certain populations, and is suspected of helping manage host responses to infection [23], [43]. Homologs of genes that flank the HP0519-*cag* PAI cluster in *H. pylori* are next to each other in both *H. cetorum* strains (e.g., HCD_05445 and HCD_05440; and HCW_05215 and HCW_05220); it is not known whether *H. cetorum* had never obtained a *cag* PAI or HP0519, vs. if this DNA segment was lost by deletion.

#### Extra urease genes

Stomach-colonizing *Helicobacter* species produce a urease that hydroylzes urea using nickel as a cofactor, and that is essential for gastric infection [44]. Remarkably several species from carnivore hosts each produce an additional urease, cofactored by iron rather than nickel [*H. acinonychis* (big cats), *H. felis* (domestic cats and dogs), and *H. mustelae* (ferrets)] [45], [46]. The two *H. cetorum* strains also contain genes for both iron- and nickel-cofactored ureases – for example, in the dolphin strain, genes HCD_02705 and HCD_02710, 94% and 97% protein level identity to *H. acinonychis ureA2* and *ureB2* (iron) and HCD_03580 and HCD_03585, ∼94% and ∼98% identity to *H. pylori ureA* and *ureB* (nickel). Equivalent homologs are found in the whale strain. Since nickel is limiting and iron is abundant in meat, an iron-cofactored urease is considered adaptive for carnivore infection [45], [46] (although *H. heilmannii sensu stricto* and *H. bizzozeronii*, which infect cats and dogs, respectively, have only a nickel-dependent urease).

#### Sel1-like repeat (*slr*) family genes

Seven and nine members of the divergent *slr* gene family, whose encoded products are secreted, and contain one or more copies of a motif characteristic of Sel1-type eukaryotic regulatory factors, were found in the dolphin and whale strain, respectively. The three best known *H. pylori* SLR proteins are: HcpA, which may modulate immune responses to infection by stimulating the release of cytokines IFN-γ, TNF-α, IL-6, IL-10 and IL-12, and differentiation of Thp1 monocytes to macrophages [47]; HcpC, which facilitates GroEL chaperone and urease translocation to the bacterial surface, and stimulates *H. pylori* growth in mammalian cell cultures [48] and also interacts with eukaryotic protein kinase Nek9 (implicated in eukaryotic cell cycle regulation) [49]; and HP0519, which, as noted above, has undergone intense selection for amino acid change in particular human populations [23], [43]. Of these, only genes closely related to *hcpC* were found in *H. cetorum* genomes (genes HCD_08435 and HCW_08325; 86% and 79% protein level identity, respectively, to closest *H. pylori hcpC* homologs), although the C terminal 150 codons of HCD_03275 and HCW_00125 exhibit ∼32% protein level identity to corresponding regions of *H. pylori* HcpA.

#### Virulence-associated *Leptospira*/*Bartonella* paralog gene family

A remarkable multigene family implicated in pathogenesis in species of *Leptospira* and *Bartonella* (PF07598; up to 12 divergent copies in the most virulent strains) [50] is represented by one distant homolog in each *H. cetorum* strain (HCW_01460 and HCD_04445). No member of this family is found in any of the many dozens of *H. pylori* strains genome sequenced to date. Just how this gene family can contribute to infection, virulence or other phenotypes that increase fitness is not yet known.

#### Outer membrane protein (OMP) genes

The *H. cetorum* strains each contain 78 or more putative OMP genes, whose various functions should include bacterial adherence to host tissues, uptake of ions, solutes and larger molecules; export of effectors and toxic metabolites, antimicrobial resistance, outer membrane assembly, etc. This gene number compares with the approximately 64 OMP genes found in annotations of *H. pylori* genomes [51, and unpublished]. A first-pass BLASTP comparison indicates that the most closely matched OMP pairs from the two *H. cetorum* strains tend to be very divergent from one another. For example, the median level of identity of whale strain OMPs to the most closely related dolphin strain homologs is only about 62%, with a range from 0% (no significant homolog) to >86% in the 35 representative proteins screened. This contrasts with the median ∼95% identity (>90% identity of some 84% of individual *H. pylori* OMPs) between unrelated *H. pylori* strains such as 26695 and J99 [51]. Superimposed on this diversity, many *H. cetorum* OMPs are more related to other OMPs in the same strain than to any homolog in the other strain; and many pairs of *H. cetorum* OMPs, although ≥80% identical in C terminal ∼200 amino acids, exhibit <30% sequence identity in their more central segments, which are likely to mediate interactions with other molecules or cells. In *H. pylori* such central region protein divergence patterns is typical of OMPs encoded by different genes, not products of strain-specific alleles of the same OMP gene. These divergences suggest OMP gene transfer from other bacterial phyla and/or different selective forces once these genes appeared in *H. cetorum* lineages, which, in turn, may have led to significantly different spectra of OMP functions in the two strains and affected cell type or host specificity.

### Competence Genes

The three separate clusters of genes needed collectively for *H. pylori* DNA transformation (genes HP0014-HP0018  =  *comB1-comB5*; HP0036-HP0042  =  *comB6-comB10*; and *dprA* and *dprB*) are present in *H. cetorum* genomes. The *comB*-encoded type IV secretion system is used in recipient cells to facilitate DNA transfer by bacterial conjugation [52]. DprA protein binds DNA and can help protect it from restriction and stimulate its methylation [53]. The presence of these genes supports ideas of DNA exchange as a force in *H. cetorum* evolution.

### Transposable Elements

Distributions of bacterial transposable elements reflect patterns of horizontal DNA transfer (genetic exchange) in populations. Three distinct classes are known in *Helicobacter*: **1**) the IS*605* family of IS elements, whose five known types are each ∼2 kb long and contain a transposase gene (*orfA*) and one or two auxiliary genes of unknown function [54]–[57]; **2**) the ∼40 kb Tn*PZ* “plasticity zone” transposons, which contain genes implicated epidemiologically in virulence in some human populations [22], and also genes for a type IV secretion system (*tfs3*) and for a novel putative integrase protein (*xerT*) [22], [58]; **3**) inducible plaque-forming prophages, found in a few East Asian *H. pylori* strains [59], [60] and remnants of them found in some other strains [14, 61, and present analyses].

The dolphin strain chromosome contains two IS*605* family members — one copy of an element closely related to IS*605* itself, plus 20 nearly identical copies of an IS*606*-type element (∼82% DNA identity to *H. pylori* IS*606*) [54]. Also present are multiple fragments of a Tn*PZ* element plus more than 20 fragments with significant matches to 1961P-type *H. pylori* phages [59], [60]. Among these are three near perfect repeats of fragments with lengths of ∼631 bp, 908 bp and 1260 bp in four, two and three locations, respectively, in the dolphin strain chromosome.

The whale strain chromosome, in contrast, lacks IS*605*-family elements, and contains two apparently complete Tn*PZ* elements, one classified as “type 2” based on gene order and 80–85% DNA identity to *H. pylori* type 2 Tn*PZ*s described in [22], and another that could be considered a type 1/type 2 hybrid or a third Tn*PZ* transposon type [22]. Also present is a 39 kb sequence that contains most genes found in the 1961P phage group (from genes HCW_02700 through HCW_02905). The first 19 kb consists of a relatively uninterrupted set of homologs of phage 1961P genes gp1 to gp18 [59] (HCW_02700 to HCW_02770), whereas the remaining ∼20 kb contain homologs of known phage genes interspersed with other (probably bacterial) genes in an order that is scrambled relative to that in 1961P and related plaque forming phages.

### Plasmids

The dolphin and whale *H. cetorum* strains contain partially related plasmids, 14.1 kb and 12.5 kb in length, respectively. Some 40% of the smaller whale strain plasmid exhibits 71%–92% DNA identity to the larger dolphin strain plasmid and contains genes implicated in plasmid DNA replication; the other 60% of this plasmid is absent by BLASTN criteria from the dolphin strain plasmid. Among features unique to the dolphin strain plasmid are (i) genes provisionally classified as encoding NTPase – DNA partitioning (HCD_08789), DNA nicking (*nikB*, HCD_08804) and DNA mobilization (*mobC*, HCD_08799) functions, which suggests that the plasmid might be readily transferred to other bacterial strains; and (ii) a direct non-tandem repeat of IS*606* elements that are nearly identical to those in the chromosome.

The fragmentation of prophages in both strains suggests ancient phage infection and lysogenization event(s); in contrast, the number and homogeneity of the dolphin strain's IS*606* elements suggests evolutionarily recent introduction and rapid copy number expansion by tranposition.

## Discussion

We sequenced the genomes of two strains of *H. cetorum*, a taxonomic group that infects marine mammals worldwide and that, based on 16 S rDNA sequences, seemed most closely related to the human gastric pathogen *H. pylori* and its derivative from big cats, *H. acinonychis*. Our genome sequences and analyses of shared genes confirm this close relationship genome-wide. That said, less than three-fourths of whale and dolphin strain genome sequences are found by BLASTN default criteria in *H. pylori* genome sequences. In addition, these strains differ remarkably from one another in: (i) sequences of many shared genes, (ii) overall content of strain-specific DNAs, and (iii) chromosomal gene arrangement. These differences are far more pronounced than are seen with strains of *H. pylori,* which is generally considered one of the most genetically diverse of bacterial species. Further studies, especially using additional *H cetorum* strains from various hosts and geographic regions are needed to learn if the two strains studied here represent different discrete groups that perhaps should be designated as separate species, vs. simply points on a genetic continuum of one extraordinarily diverse species. In considering this issue, we note that the traditional species concept as developed for higher organisms is poorly suited to bacteria. This is because many bacterial phyla have rich histories of DNA transfer from unrelated groups, superimposed on reproduction by clonal growth without need for gene exchange [62].

Multiple features distinguish the genomes of these *H. cetorum* strains from those of *H. pylori* and *H. acinonychis,* most prominently: (i) their positions in a phylogenetic tree based on sequences of shared core genes (Figure 1); and (ii) the 36% of the whale strain and 26% of the smaller dolphin strain genomes not found in *H. pylori* genomes by Mega BLASTN criteria. Such features suggest *H. cetorum* genome evolution driven by horizontal DNA transfer from other phyla, in addition to *in situ* mutation, selection for adaptive change and genetic drift. Supporting this view are differences in metabolic enzymes illustrated in Figures 3 and 4; OMPs and other proteins likely to participate directly in bacterial host interaction; and contents of mobile DNAs (the IS*605*-family elements, Tn*PZ* transposons and prophage remnants). We note, in particular the differences in ∼80 putative outer membrane proteins, many of which may participate in adherence and signaling to host tissues, uptake or export of ions and molecules, and membrane synthesis (Tables 3 and 4); and also the remarkably divergent alleles of the *vacA* (vacuolating cytotoxin) gene in the usual location next to *cysS* and in the dolphin strain's extra triplet of *vacA* genes inserted nearby (Figure 5). The most intense divergence among the various *H. cetorum* VacA proteins is in the first ∼700–800 amino acids, which in well characterized VacA proteins, contains a signal sequence needed for VacA secretion and determinants of the protein's multiple host cell intoxication activities [32]–[35]. Future studies may reveal novel functionalities of these various *vacA* alleles, how their divergent sequences affect the transport, actions and interactions of their encoded proteins, and the selective forces that drive their evolution.

Metabolic differences also merit particular attention: Prominent among them are *H. cetorum*'s rhodonase sulfurtransferase, which may catalyze synthesis of pyruvate and thiosulfate from 3-mercaptopyruvate (Figure 3; blue arrows). These sulfurtransferases are related to enzymes found in diverse genera including *Haemophilius* and *Actinobacillus*, but in few if any other members of the *Epsilonproteobacteria*. A second example is provided by *H. cetorum*'*s* distinctive NADP-dependent malic enzyme, which should catalyze production of L-malate from pyruvate (Figure 4, blue arrows), and whose homologs occur in multiple extragastric *Helicobacter spp*, but not in *H. pylori*. Also noteworthy are the metabolic enzymes found in *H. pylori* but not *H. cetorum*: in particular those for synthesis of L-homocysteine and conversion of L-cysteine to thiocysteine or pyruvate (Figures 3; red arrows); and those for syntheses of acetoacetyl-CoA and acetate from acetyl-CoA, and of acetoacetate from acetoacetyl-CoA (Figure 4; red arrows). Finally we note the phosphoenolpyruvate carboxylase (production of oxaloacetate from phosphoenolpyruvate) in the whale but not the dolphin strain (Figure 4; green arrow). Although direct experimental analyses are needed to fully understand these enzymes and their actions and importance *in vivo*, our findings fit with a suggestion, made while describing *H. bizzozeronii* [31], that *Helicobacter* adaptation to particular hosts could in part involve acquisition or loss of specific metabolic pathways,

Many additional features of interest to particular readers will be found in our two *H. cetorum* genome sequences, which should also aid further analyses of issues such as: (i) this species' great diversity and how these microbes have adapted for chronic infection of their various marine mammal hosts; (ii) how genetically interconnected or separate *H. cetorum* populations from different oceans or host species may be; (iii) mechanisms of *H. cetorum* transmission within and among host species; (iv) host ranges and factors that determine host specificity; (v) the relative importance for *H. cetorum* strain genetic divergence of mutation and horizontal gene transfer, and of selection for adaptive change and genetic drift (e.g., due to specialization for different host species or the vastness of the world's oceans); and (vi) finally the pathogenic vs. benign or beneficial interactions of *H. cetorum* strains with their various hosts, an issue of particular interest in today's fragile marine ecosystems.

## Supporting Information

Table S1 *Annotation from *H. pylori* 26695 NCBI BioProject PRJNA178201.
(DOCX)
