# Supplementary material for: Sequence Divergence and Conservation in Genomes of Helicobacter cetorum Strains from a Dolphin and a Whale
Source: PLoS One. 2013 Dec 17;8(12):e83177. doi: 10.1371/journal.pone.0083177 (PMC3866246; doi:10.1371/journal.pone.0083177)
Supplement: Table S1 — *Annotation from H. pylori 26695 NCBI BioProject PRJNA178201. (DOCX) [file pone.0083177.s001.docx]

Table S1. Core genes of *H. pylori* and *H. cetorum*, also present in the non-gastric species *H. hepaticus* used for phylogenetic analysis (Figure 1).

| Locus_tag | GOs | Annotation |
| --- | --- | --- |
| HP0055 | HEL1079 | Sodium/proline symporter |
| HP0056 | HEL1080 | Delta-1-pyrroline-5-carboxylate dehydrogenase |
| HP0074 | HEL1081 | Lipoprotein signal peptidase (*ispA*) |
| HP0075 | HEL1082 | Phosphoglucosamine mutase (*glmM*) |
| HP0076 | HEL1083 | 30S Ribosomal protein S20 (*rpsT*) |
| HP0077 | HEL1084 | Peptide chain release factor 1 (*prfA*) |
| HP0083 | HEL1085 | 30S Ribosomal protein S9 (*rpsI*) |
| HP0084 | HEL1086 | 50S ribosomal protein L13 (*rplM*) |
| HP0090 | HEL1087 | ACP S-malonyltransferase |
| HP0145 | HEL1089 | *cbb3*-Type cytochrome c oxidase subunit II |
| HP0149 | HEL1090 | Hypothetical protein |
| HP0152 | HEL1091 | Hypothetical protein |
| HP0173 | HEL1092 | Flagellar biosynthesis protein (*fliR* ) |
| HP0178 | HEL1093 | sialic acid synthase |
| HP0197 | HEL1094 | S-adenosylmethionine synthetase |
| HP0198 | HEL1095 | Nucleoside diphosphate kinase (*ndk*) |
| HP0199 | HEL1096 | Hypothetical protein |
| HP0202 | HEL1097 | 3-Oxoacyl-ACP synthase |
| HP0210 | HEL1098 | Heat shock protein 90 |
| HP0212 | HEL1099 | Succinyl-diaminopimelate desuccinylase |
| HP0224 | HEL1100 | Bifunctional methionine sulfoxide reductase A/B |
| HP0231 | HEL1101 | Hypothetical protein |
| HP0237 | HEL1102 | Porphobilinogen deaminase (*hemC*) |
| HP0238 | HEL1103 | Prolyl-tRNA synthetase |
| HP0239 | HEL1104 | Glutamyl-tRNA reductase (*hemA*) |
| HP0240 | HEL1105 | Octaprenyl-diphosphate synthase (*ispB*) |
| HP0241 | HEL1106 | Hypothetical protein |
| HP0242 | HEL1107 | Hypothetical protein |
| HP0243 | HEL1108 | Neutrophil activating protein (*napA*) (bacterioferritin) |
| HP0264 | HEL1110 | ATP-dependent protease binding subunit (*clpB*) |
| HP0284 | HEL1111 | HHP0284 hypothetical protein |
| HP0285 | HEL1112 | Hypothetical protein |
| HP0286 | HEL1113 | Cell division protein (*ftsH*) |
| HP0318 | HEL1115 | Hypothetical protein |
| HP0319 | HEL1116 | Arginyl-tRNA synthetase (*argS*) |
| HP0321 | HEL1117 | Guanylate kinase (*gmk*) |
| HP0323 | HEL1118 | Nuclease (*nucT*) |
| HP0325 | HEL1119 | Flagellar basal body L-ring protein (*flgH*) |
| HP0328 | HEL1120 | Tetraacyldisaccharide 4'-kinase (*lpxK*) |
| HP0329 | HEL1121 | NAD synthetase |
| HP0385 | HEL1122 | Hypothetical protein |
| HP0409 | HEL1124 | GMP synthase (*guaA*) |
| HP0466 | HEL1125 | Hypothetical protein |
| HP0469 | HEL1126 | Hypothetical protein |
| HP0470 | HEL1127 | Oligoendopeptidase F (*pepF*) |
| HP0480 | HEL1128 | GTP-binding protein |
| HP0487 | HEL1129 | Hypothetical protein |
| HP0581 | HEL1131 | Dihydroorotase |
| HP0584 | HEL1132 | Flagellar motor switch protein |
| HP0585 | HEL1133 | Endonuclease III (*nth*) |
| C694_03020* | HEL1134 | Ferrous iron transport protein A |
| HP0586 | HEL1135 | Hypothetical protein |
| HP0587 | HEL1136 | Aminodeoxychorismate lyase |
| HP0588 | HEL1137 | 2-Oxoglutarate-acceptor oxidoreductase subunit (*oorD*) |
| HP0590 | HEL1138 | 2-Oxoglutarate-acceptor oxidoreductase subunit (*oorB*) |
| HP0599 | HEL1139 | Hemolysin secretion protein precursor (*hylB*) |
| HP0602 | HEL1140 | 3-Methyladenine DNA glycosylase |
| HP0604 | HEL1141 | Uroporphyrinogen decarboxylase (*hemE*) |
| HP0605 | HEL1142 | Hypothetical protein |
| HP0606 | HEL1143 | Membrane fusion protein (*mtrC*) |
| HP0607 | HEL1144 | Acriflavine resistance protein (*acrB*) |
| HP0615 | HEL1145 | NAD-dependent DNA ligase (*ligA*) |
| HP0616 | HEL1146 | Chemotaxis protein (*cheV*) |
| HP0617 | HEL1147 | Aspartyl-tRNA synthetase (*aspS*) |
| HP0618 | HEL1148 | Adenylate kinase (*adk*) |
| HP0620 | HEL1149 | Inorganic pyrophosphatase |
| HP0640 | HEL1150 | Poly(A) polymerase |
| HP0654 | HEL1152 | Hypothetical protein |
| HP0656 | HEL1153 | Hypothetical protein |
| HP0661 | HEL1154 | Ribonuclease H (*rnhA*) |
| HP0662 | HEL1155 | Ribonuclease III (*rnc*) |
| HP0663 | HEL1156 | Chorismate synthase |
| HP0664 | HEL1157 | Hypothetical protein |
| HP0665 | HEL1158 | Coproporphyrinogen III oxidase |
| HP0666 | HEL1159 | Glycerol-3-phosphate dehydrogenase |
| HP0680 | HEL1160 | Ribonucleotide-diphosphate reductase subunit alpha |
| HP0687 | HEL1161 | Iron(II) transport protein (*feoB*) |
| HP0711 | HEL1162 | Hypothetical protein |
| HP0715 | HEL1163 | ABC Transporter ATP-binding protein |
| HP0727 | HEL1164 | Transcriptional regulator |
| HP0745 | HEL1165 | Hypothetical protein |
| HP0746 | HEL1166 | HHP0746 hypothetical protein |
| HP0747 | HEL1167 | XtRNA (guanine-N(7)-)-methyltransferase (*rnc*) |
| HP0748 | HEL1168 | Cell division protein (*ftsE*) |
| HP0750 | HEL1169 | Hypothetical protein |
| HP0751 | HEL1170 | Flagellar protein (*flaG)* |
| HP0752 | HEL1171 | Flagellar capping protein (*fliD*) |
| HP0753 | HEL1172 | Flagellar protein (*fliS*) |
| HP0763 | HEL1173 | Cell division protein (*ftsY*) |
| HP0831 | HEL1174 | Dephospho-CoA kinase (*coaE*) |
| HP0835 | HEL1175 | Histone-like DNA-binding protein HU (*hup*) |
| HP0842 | HEL1176 | Hypothetical protein |
| HP0853 | HEL1178 | ABC transporter ATP-binding protein |
| HP0875 | HEL1179 | Catalase |
| HP0907 | HEL1180 | Flagellar basal body rod modification protein (*flgD*) |
| HP0920 | HEL1181 | Hypothetical protein |
| HP1025 | HEL1182 | Heat shock protein (*hspR*) |
| HP1027 | HEL1183 | Ferric uptake regulation protein |
| HP1029 | HEL1184 | Hypothetical protein |
| HP1043 | HEL1185 | Response regulator |
| HP1076 | HEL1186 | Hypothetical protein |
| HP1123 | HEL1188 | Peptidyl-prolyl cis-trans isomerase, FKBP-type rotamase (*slyD*) |
| HP1124 | HEL1189 | Hypothetical protein |
| HP1125 | HEL1190 | Peptidoglycan associated lipoprotein precursor (*omp18*) |
| HP1189 | HEL1191 | Aspartate-semialdehyde dehydrogenase |
| HP1242 | HEL1194 | Hypothetical protein |
| HP1275 | HEL1196 | Phosphomannomutase (*algC*) |
| HP1277 | HEL1197 | Tryptophan synthase subunit alpha (*trpA*) |
| HP1281 | HEL1198 | anthranilate synthase component II |
| HP1282 | HEL1199 | Anthranilate synthase component I |
| HP1346 | HEL1200 | Glyceraldehyde-3-phosphate dehydrogenase |
| HP1348 | HEL1201 | 1-Acylglycerol-3-phosphate O-acyltransferase |
| HP1349 | HEL1202 | Hypothetical protein |
| HP1350 | HEL1203 | Protease |
| HP1391 | HEL1206 | Hypothetical protein |
| HP1429 | HEL1207 | Polysialic acid capsule expression protein (kpsF) |
| HP1430 | HEL1208 | ATP-binding protein |
| HP1431 | HEL1209 | Dimethyladenosine transferase (*ksgA*) |
| HP1496 | HEL1214 | 50S Ribosomal protein L25 |
| HP1497 | HEL1215 | Peptidyl-tRNA hydrolase |
| HP1498 | HEL1216 | Hypothetical protein |
| HP1514 | HEL1217 | Transcription elongation factor (*nusA*) |
| HP1523 | HEL1218 | ATP-dependent DNA helicase (*recG*) |
| HP1532 | HEL1219 | Glucosamine--fructose-6-phosphate aminotransferase |
| HP1554 | HEL1220 | 30S Ribosomal protein S2 (*rpsB*) |
| HP1555 | HEL1221 | elongation factor Ts (*tsf*) |
| *Annotation from *H. pylori* 26695 NCBI BioProject PRJNA178201 | | |
